# Airborne Transducer Integrity under Operational Environment for Structural Health Monitoring

**DOI:** 10.3390/s16122110

**Published:** 2016-12-12

**Authors:** Mohammad Saleh Salmanpour, Zahra Sharif Khodaei, Mohammad Hossein Aliabadi

**Affiliations:** Department of Aeronautics Imperial College, London SW7 2AZ, UK; z.sharif-khodaei@imperial.ac.uk (Z.S.K.); m.h.aliabadi@imperial.ac.uk (M.H.A.)

**Keywords:** temperature, vibration, Lamb wave, composites, non-destructive testing

## Abstract

This paper investigates the robustness of permanently mounted transducers used in airborne structural health monitoring systems, when exposed to the operational environment. Typical airliners operate in a range of conditions, hence, structural health monitoring (SHM) transducer robustness and integrity must be demonstrated for these environments. A set of extreme temperature, altitude and vibration environment test profiles are developed using the existing Radio Technical Commission for Aeronautics (RTCA)/DO-160 test methods. Commercially available transducers and manufactured versions bonded to carbon fibre reinforced polymer (CFRP) composite materials are tested. It was found that the DuraAct transducer is robust to environmental conditions tested, while the other transducer types degrade under the same conditions.

## 1. Introduction

Permanently attached transducer networks are an important part of structural health monitoring (SHM) systems. These systems can be used for damage detection in carbon fibre reinforced polymer (CFRP) composite materials in aircraft structures. Typical airliners operate in a range of conditions, hence SHM transducer integrity must be demonstrated for this range of environments.

Classical non-destructive inspection methods are an integral part of the current maintenance schedule, SHM systems are accepted as being in the development stage. The International Maintenance Review Board Policy Board (IMRBPB) [1] set out definitions for Scheduled-SHM, the authors of [2] made further additions with Automated-SHM defined in general terms. The distinction being A-SHM is any SHM technology without a pre-determined interval for structure maintenance actions, instead relying on the system to inform when action must take place.

SHM covers a wide range of approaches employing various sensor/actuator types. In guided wave SHM a network of attached ultrasonic transducers, typically piezoelectric Lead Zirconate Titanate (PZT), generate and sense diagnostics signals. Several promising systems have been demonstrated under laboratory conditions [3,4,5,6,7,8]. Researchers have investigated the effects of temperate [9,10,11,12,13], humidity [14] and vibration loading [15] on damage detection systems. The main focus of these works has been to assess environmental influences on pristine and damage propagation features. While no set framework exist for certification and testing of SHM systems, Federal Aviation Administration (FAA) recommendations [16,17] and existing standards [17] can provide a foundation. The latter makes references to transducer and connection network integrity assessments through Radio Technical Commission for Aeronautics (RTCA) DO-160 Environmental Conditions and Test Procedures for Airborne Equipment and US Military Standard MIL-STD 810 [18,19].

This paper investigates robustness requirements for permanently attached PZT transducers. It presents results for test profiles tailored to the environmental conditionals that an SHM system will be exposed to during operation using existing frameworks: RTCA/DO-160C and MIL-STD 810. Tests conditions for a regional aircraft in Europe were performed for airborne transducers, connection network and attachment on four sample types: DuraAct, DuraAct with Kevlar coating, Smart Layer and a manufactured SHM layer. These tests included high and low temperature, thermal shock, altitude and vibration. It was found that the DuraAct transducer outperformed in the integrity tests and was the most reliable.

## 2. Environmental Conditions and Parameters

As the components to be tested are permanently attached to a host structure, they will become airborne at some part of their operational life. S-SHM would then be performed on the ground. Assessing the performance of an A-SHM system while airborne is not the focus of this paper. It is assumed that the host can perform structurally as designed.

The following assumptions about the aircraft will serve as key factors in determining the operation environment and hence the required test profile: fixed wing subsonic, turbofan propulsion, maximum operating altitude of 50,000 ft and regional aircraft operating in Europe. In addition it is assumed that transducers and connection network will be: internally mounted, in a non-temperature controlled area, in a non-pressure controlled area and not in extreme temperature locations, e.g., engine cowling or exhaust outlets.

It should be noted that for DO-160 ambient conditions can be within the range: temperature 25 ± 10 °C; humidity <85% RH and pressure 84 to 107 kPa. During the test, conditions must have stabilised: pressure ±5% and temperature ±3 °C. The tests performed are summarised in Table 1.

**Low and High Temperature:** MIL-STD 180 gives the extreme temperature in Europe as −55 and 50 °C in Ust’Shchugor, Russia and Seville, Spain respectively. Additionally, the DO-160C defines the low temperature as −55 °C and high as 70 °C which is the same or exceeds Europe’s extreme temperatures. The temperature will be reduced or increased from ambient, then held constant for three hours at the low or high temperatures receptively. Temperature ramp rate is not defined in test category D0-160C 4.5.1,3 as these are not intended to be dynamic temperature tests.

**Figure 1:**
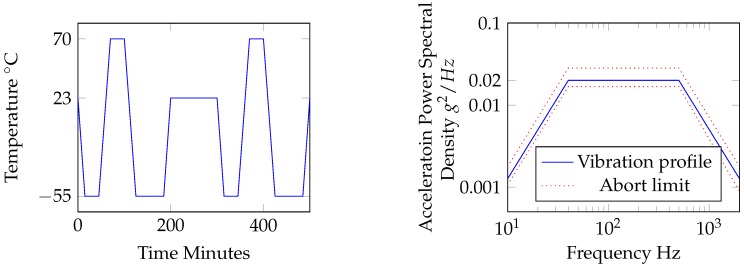
Test profiles for thermal shock (**left**) and random vibration profile (**right**), slops ±6 dB/octave.

**Thermal Shock/Temperature Variation:** Components may experience temperature variations during normal operation e.g., during take-off an landing. This test is intended to be a dynamic temperature test. For internally mounted equipment in non-temperature controlled sections of the aircraft DO-160 defines a ramp rate of 5 °C/min. Temperature will be ramped between the extreme high and low temperatures and repeated twice, as shown in Figure 1 (left).

**Altitude:** During normal operation the components in non-pressurised locations will encounter changes in pressure from ambient (sea/ground level) to cruise level. The maximum operation altitude was set at 50,000 feet with a pressure of 11.6 kPa (absolute), and maintained for 2 h. To de-couple the effect of pressure and temperature category DO-160 4.6 specifies ambient temperature for this test.

**Vibration:** Depending on equipment location DO-160 specifies a certain vibration regime and level. For a turbojet (and turbofan) standard random tests must be performed with the sample fixture representative of the actual operational structure. The random acceleration power spectral density (APSD) for a fuselage location was chosen, as shown in Figure 1 (right).

### Integrity and Robustness Parameters

Ultrasonic guided wave propagation in thin plate like structures are sensitive to the material properties and boundary conditions [20]. This is exploited to detect structural damage and flaws [6,7]. Wave propagation is sensitive to the condition of the transducer and connection network. In the absence of changes to the host material properties, any degradation in the components will be apparent as a residual between signals obtained before and after the degradation.

The transducer integrity under each environmental profile will be assessed by comparing voltage-time signals B[t] obtained before the test, with those for after the test A[t], where *t* is time. The difference D[t] can be found as D[t]=B[t]−A[t]. The envelope *E* is then calculated as the magnitude of the analytical signal:

(1) $$\begin{array}{c} E[t]=|D[t]+iH(D[t])| \end{array}$$

where H is the Hilbert transform [21].

Only the first segment of the signal is considered, consisting of the first four wave packets ($0⩽t⩽t_{4}$). The initial segment was chosen as this part of the signal is critical for damage localisation and also to help mitigate any effects of boundary conditions that may change during the test [6,22]. The integrity pass-threshold is closely related to the false alarm rates, probability of detection and detectable damage size; this is chosen such that the minimum damage scatter to baseline ratio is 10% or −20 dB [23]. The sample will be deemed to have maintained integrity if the envelope peak is under one tenth of the maximum signal amplitude e.g., residual index *R* is less than 10%:

(2) $$R=\frac{max(E[t])}{max(D[t])}\times 100,\;for\;0⩽t⩽t_{4}$$

## 3. Experimental Set-Up

Ultrasonics signals prior, during and after each test were generated and recorded using a National Instruments (NI) platform, with each transducer used as actuator and sensor (pitch catch). This was done using a PXIe 5412 arbitrary voltage generator, PXIe 5105 digital oscilloscope and a Pickering 40–726 A switching card with maximum output voltage amplitude of 12 volts. Ultrasonic Lamb waves were excited with a five cycle Hanning tone-burst with central frequency swept in the range of 50–350 kHz. This ensured signals were available at both low (50 kHz) and high (300 kHz) frequencies with dominant $A_{0}$ and $S_{0}$ modes respectively. The response was sampled at 60 MS/s for 0.001 s. Each recording was repeated 10 times, bandpass filtered and averaged.

### 3.1. Vibration Set-Up

**Figure 2:**
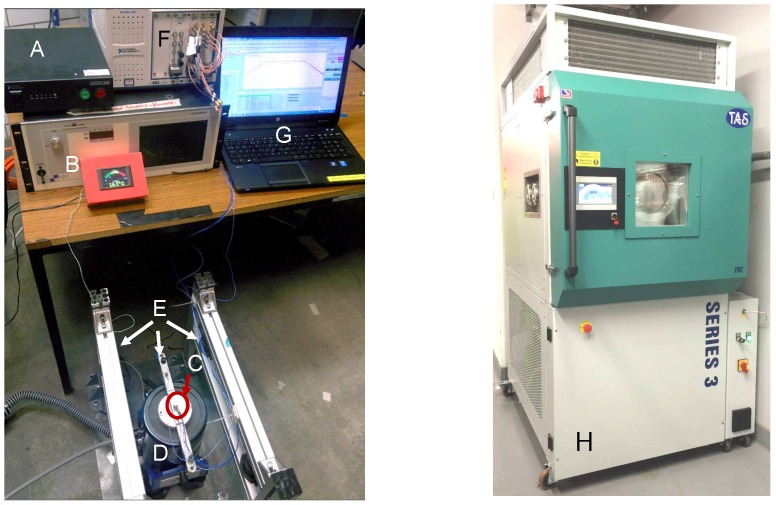
(**A**) Controller; (**B**) Shaker power amplifier; (**C**) Control accelerometer; (**D**) Shaker; (**E**) Fixture; (**F**) National Instruments (NI) signal generation and sensing platform; (**G**) Workstation and (**H**) Test chamber.

The vibration set-up consisted of a TMS 2110E shaker driven with a 2050E09-FS power amplifier. This was controlled with a Crystal Instruments Spider 81-B control and acquisition unit. A PCB Piezotronics 352C33 high sensitivity accelerometer was used for the control measurements on the sample. Three fixture clamps were 15 cm apart, replicating stiffener bays, with the shaker coupling at the centre shown in Figure 2. Plate vibration was in the out of plane direction. A frequency response function was recorded for each sample which was used in a feedback loop to achieve the required APSD profile.

### 3.2. Thermal and Altitude Set-Up

The samples were exposed to the environmental profiles with a TAS Series 3 temperature, climatic and pressure test chamber, shown in Figure 2H manufactured by TAS Ltd., Goring-By-Sea, UK [24]. The chamber temperature was measured with a Platinum resistance thermometer probe, and on each sample with K-type thermocouples. Sample temperatures were recorded using an NI cDAQ 9211 thermocouple module. Temperature and humidity output readings of the chamber were continually recorded during the tests. Sample temperatures were used as the target for temperature profiles. The chamber absolute pressure was reduced to simulate pressure levels at 50,000 ft. KF-50 pressure fittings were used for coaxial and thermocouple signal pass-through in to the pressurised test chamber.

Each sample was conditioned by maintaining at 70 °C for three hours. This was followed by exposure and dwell between maximum and minimum temperatures, this was repeated at least 10 times.

### 3.3. Samples

**Figure 3:**
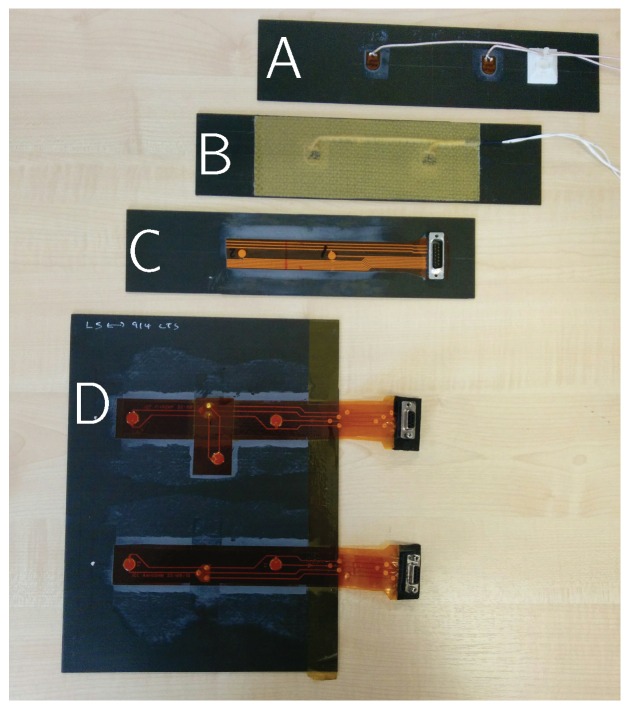
(**A**) DuraAct; (**B**) Co-Bonded; (**C**) Smart Layer; and (**D**) structural health monitoring (SHM) layer.

Four transducer types were tested for integrity by mounting on CFRP host plates. All samples were made from the same host material consisting of 16 unidirectional Hexply 914-TS-5-134 plies with stacking sequence [0, 45, −45, 90]${}_{2s}$ of 2 mm overall thickness. The transducers were bonded to the top surface with Hexcel Redux 312 film adhesive as shown in Figure 3.

An advantage of the Smart Layer and SHM layer is that the connection network is contained within the layer itself. However, for DuraAct transducers, wires must be soldered separately and secured individually. The Kevlar layer was applied only for DuraAct transducers as this could provide a layer of protection for solder connections and to secure the wiring. While tear and penetration resistance of Kevlar may have also been beneficial for the Smart Layer and SHM layer, mechanical testing was not the focus of this study.

**Figure 4:**
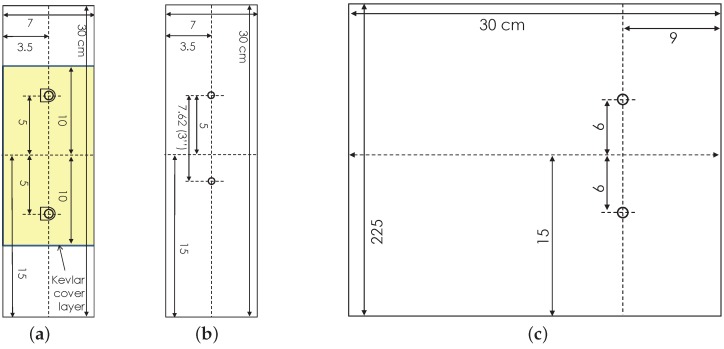
(**a**) Sample 1 without Kevlar layer and Sample 2 with Kevlar layer; (**b**) Sample 3 (Smart Layer) schematic; (**c**) Sample 4 (SHM layer) schematic.

**Sample 1 (DuraAct):** Two PIC DuraAct transducers (manufactured by PIC [25]) were mounted centrally as shown in Figure 4a. Each of these had PZT discs 10 mm × 0.2 mm (diameter × thickness) potted in proprietary resin covered with polyimide (Kapton) film, giving overall dimension of 17 mm × 13 mm. Transducers soldered and electrically connected with RG178 cable (MIL-C-17G).

**Sample 2 (Co-bonded Kevlar):** This had identical transducer layout to sample 1, but with a Kevlar protective layer and 35% thinner RGW 5274 coaxial cable, see Figure 4a. The transducers, film adhesive and the Kevlar prepreg (MTM28-48% -K49127-4H-170) were co-bonded to the host in a vacuum-bag process at 120 °C.

**Sample 3 (Smart Layer):** This consisted of multiple PZT discs (1/4" diameter) with a flexible connection network sandwiched between polyimide film (manufactured by Acellent Technologies Inc. Sunnyvale, (CA, USA) [26]), see Figure 4b. The PZT discs were attached to the layer with unspecified resin leaving the bottom electrode exposed. The layer and the bottom surface of the PZTs discs were bonded on the host using the film adhesive. The layer used a D-Sub to coaxial connector and cables terminated with BNC connectors.

It must be noted that the Smart Layer was found to be the most fragile before and during the attachment stage. Multiple layers failing before attachment as the exposed transducer discs separated from the layer.

**Sample 4 (SHM Layer):** The SHM layer was manufactured in-house as a composite layup. This consisted of double sided flex circuit and PZT discs sandwiched in Kapton film with B-staged modified acrylic adhesive. The discs (diameter × thickness 10 mm × 0.25 mm) were position 120 mm apart and covered with Kapton film on both sides, see Figure 4c. A D-sub connector were used with RG178 cable.

## 4. Results and Discussion

The samples were exposed to the test profiles and their integrity assesses after the tests. The samples were visually inspected after each test, and in all cases no visible degradation was observed. Test results are summarised in Table 2.

**Low Temperature:** the Smart Layer displayed the most degradation with all frequencies effected. The degradation in the Smart Layer could have been due to the unspecified epoxy adhesive used by the manufactures to attach the transducers to the polyimide layer, degrading at reduced temperatures.

**High Temperature:** the Smart Layer degraded in such a way that effected the lower frequency (A0) modes the most. While the co-bonded layer also displayed a similar trend but only 50 kHz signal was effected.

**Temperature Variation and Thermal Shock:** there was degradation in the Smart Layer when exposed to the thermal shock environment with all frequencies effected. For the manufactured SHM layer only 300 kHz signal was effected. In the co-bonded layer only 50 kHz signal was effected.

**Altitude:** the manufactured SHM layer performed particularly badly with high frequencies effected. This could be attributed to the manufacturing process of the layer, as there were sealed air pockets within the layer itself. This would put the layer under strain when the pressure is reduced, which may have caused a deterioration in the layer.

**Vibration:** all samples were robust, except the Smart Layer at 50 kHz. It must be noted that the Smart Layer connector supplied may not be suitable for in the field applications. The D-Sub connector could not be secured with the thumb screws as they were too short, and the connector itself was not positive latching.

## 5. Conclusions and Future Work

A set of integrity tests based on the RTCA/DO-160 Environmental Conditions and Test Procedures for Airborne Equipment were applied to four types of permanently attached PZT transducers. Thermal, altitude and vibration operational environment profiles were applied. DuraAct transducers maintained integrity after all of these tests. The SHM layer was found to degrade the most when exposed to the altitude environment. Degradation in the Smart Layer was apparent from the higher residual when exposed to the thermal environments. This may be attributed to the degradation in the unspecified epoxy resin used by the manufacturer. The SHM layer integrity was the same as or exceeded the Smart Layer integrity in all but the altitude test environment.

It must be noted that the degradation observed can only be associated to the transducer or associated connection network, not to the CFRP host, film adhesive used for attached or data acquisition system. This is because the environmental conditions were within the operating range of the host and film adhesive, and more significantly the DuraAct sample was found to be robust to all the conditions. Future work will focus on other environmental conditions including humidity and combination thereof.

## Figures and Tables

**Table 1:** Environmental test matrix. Sample type definitions in Section 3.3.

Test Category	Environment	Certification Category	Samples Types
Low Temperature	−55 °C	DO-160 C 4.5.1	4
High Temperature	70 °C	DO-160 C 4.5.3	4
Thermal Shock	−55 to 70 °C at 5 °C/min	DO-160 C 5	4
Altitude	50,000 ft (11.6 kPa)	DO-160 C 4.6	4
Vibration	Random APSD 10–2000 kHz	DO-160 C 8	4

**Table 2:** Robustness and integrity results for environmental tests. Pass indicates integrity at all frequencies in range. * integrity in all but one frequency in bracket. Low *f*: 50, 75, 100, 150; High *f*: 200, 250, 300 kHz.

Condition		DuraAct	Co-Bonded Kevlar	Smart Layer	SHM Layer
Temperature Low	High *f*	Pass	Pass	Fail	Pass
	Low *f*	Pass	Fail	Fail	Pass
Temperature High	High *f*	Pass	Pass	Pass	Pass
	Low *f*	Pass	Pass * (50)	Fail	Pass
Altitude	High *f*	Pass	Pass	Pass	Fail
	Low *f*	Pass	Pass * (50)	Pass	Pass
Vibration	High *f*	Pass	Pass	Pass	Pass
	Low *f*	Pass	Pass	Pass * (50)	Pass
Thermal Shock		Pass	Pass * (50)	Fail	Pass * (300)
